# Patients arriving by ambulance to the Emergency Department; vital signs and 30 day mortality

**DOI:** 10.1186/1757-7241-19-S2-P37

**Published:** 2012-04-16

**Authors:** Frida Johansson, Charlotte Annerud, Ole Nørgaard Jensen, Annmarie Lassen

**Affiliations:** 1 University of Southern Denmark, Medical Emergency Department, Odense University Hospital, Denmark; 2 Medical Emergency Department, Åbenrå Hospital, Denmark; 3 Medical Emergency Department, Odense University Hospital, Denmark

## Background

Vital signs outside the normal range on arrival to an emergency department are related to a poor prognostic outcome. However, the knowledge of the prognostic value of vital signs measured in the ambulance is scarce.

The aim of the study was to describe vital signs measured in the ambulance (saturation, systolic blood pressure, pulse, respiratory frequency and Glasgow Coma Scale (GCS)) and their relation to 30 day mortality for patients arriving by ambulance to the Odense University Hospital (OUH) emergency department.

## Methods

A cohort study was conducted on patients arriving by ambulance to the OUH emergency department between 1st and 24th of November 2010. Data was collected from ambulance records, OUH electronic health record and the Patient Administrative system of Funen.

## Results

705 ambulance records were identified, covering 631 persons who were included at the first contact. 45% were men and the mean age was 54 years (interquartile range 29-73). 10 men and 23 women died within 30 days with a mortality rate of 35/1000 person months [95%CI 0.025-0.049] for men and 67/1000 person months [95%CI 0.053-0.085] for women.

Relative risk (RR) for 30 day mortality among patients with saturation < 90% in the ambulance was 6.95 [95% CI 3.44-14.06], 4.44 [95% CI 1.69-11.53] for systolic blood pressure < 100 mmHg, 2.04 [95% CI 0.99-4.18] for pulse ≥ 100/min, 4.29 [95% CI 2.06-8.95] for respiratory frequency ≥ 20/min and 8.99 [95% CI 2.06-8.95] for GCS < 15.

If the patient had one or more abnormal vital signs measured in the ambulance RR the 30 day mortality was 9.02 [95% CI 2.76-26.48] compared to normal range vital signs.

The registered abnormal vital signs in the ambulance improved to normal in most cases before arriving at the emergency department.

## Conclusion

Except for pulse, all abnormal vital signs measured in the ambulance were related to an increased risk of 30 day mortality.

